# Cutaneous side effects of combination therapy of pegylated interferon 2α and ribavirin

**DOI:** 10.1186/1471-2334-14-S7-P23

**Published:** 2014-10-15

**Authors:** Anca Raducan, Sorin Rugină

**Affiliations:** 1 2nd Dermatology Clinic, Colentina Clinical Hospital, Bucharest, Romania; 2 Infectious Diseases Hospital, Constanta, Romania; 3 Faculty of Medicine, “Ovidius” University, Constanța, Romania

## Background

Hepatitis C virus infection is the main cause of chronic liver disease, and patients are at risk of developing liver cirrhosis and hepatocellular carcinoma. The treatment in CHC is the combination of pegylated interferon 2α (peg-IFN) and ribavirin (RBV), the standard therapy with 24 to 48 weeks regimen. Cutaneous adverse effects are common, with a higher incidence in combination therapy than in IFN alone, and when serious, discontinuation of therapy is mandatory.

## Case report

We report the case of a woman who developed an exanthematous drug eruption during peg-IFN and RBV combination therapy for CHC. The patient was clinically examined and we performed routine tests and PCR for HCV-RNA.

A 54-year-old woman with chronic hepatitis C (HCV viral load of 1,432,722 IU/mL), presented with generalized rash and pruritus, which appeared on the upper extremities and trunk after receiving her eighth weekly injection of peg-IFN 2α (180 mcg/week), in combination with daily oral RBV (1000 mg/day) and spread to the face, palms and lower extremities.

Dermatological examination revealed an erythematous maculopapular eruption, symmetrically distributed over the trunk, extremities, cheeks and ears, associating moderate pruritus and xerosis. On her left thigh, the patient presented an ill-defined, slightly pruritic, erythematous round patch, localized to the point of administration. On both palms and fingers, there were multiple symmetric, firm, deep-seated vesicles, with intense pruritus.

The patient was treated with desloratadinum and methylprednisolone aceponate ointment 0.1%, for 10 days, with complete clearance of the eruption and cessation of pruritus. The localized patch at the site of injection healed within a month with diffuse desquamation and mild postinflammatory pigmentation. During follow-up, the patient presented with new erythematous patches at the administration point of peg-IFN. The patient reported lacking appetite, feeling weak, nauseous and dizzy.

After 12 weeks treatment, ALT and AST, with initially high values, normalized. However, considering the low virologic response (viral load 57,126 IU/mL), the patient’s overall clinical status, low prothrombin index, low body mass index and significant blood count abnormalities (anemia, thrombocytopenia and neutropenia), we decided to discontinue therapy, without new lesions at follow-up.

## Conclusion

The dermatological diagnosis was exanthematous drug eruption, which was considered a side effect of combination therapy with peg-IFN and RBV, due to the time link between the development of lesions and the CHC therapy, lack of other cause of eruption, and complete resolution after treatment withdrawal. Furthermore, the patient is still monitored and we consider other treatment options.

